# Breaking the Cycle: Investigating Family Resilience as a Pathway to Better Adolescent Mental, Emotional, Developmental, and Behavioral Health Outcomes

**DOI:** 10.1007/s40653-025-00792-0

**Published:** 2025-11-08

**Authors:** Mounika Polavarapu, Shipra Singh, Mohini Adhikari

**Affiliations:** The University of Toledo, Toledo, OH United States

**Keywords:** Adverse childhood experiences (ACEs), Family resilience, Adolescent mental health, Mental emotional, and behavioral health outcomes (MEDBH), Structural equation modeling

## Abstract

Over 20% of children aged 3–17 in the U.S. have at least one diagnosed mental, emotional, developmental, or behavioral health (MEDBH) condition. Adverse Childhood Experiences (ACEs) are linked to poor MEDBH outcomes. While family resilience has been examined as a protective factor for specific mental health outcomes, few studies have investigated its role as a distinct mediator in adolescent MEDBH. This study investigated the relationship between ACEs, family resilience, and MEDBH outcomes in adolescents aged 12–17 years. This analysis utilized cross-sectional data from the 2022 National Survey of Children’s Health (NSCH). Logistic regression models assessed the association between ACEs, family resilience, and MEDBH outcomes. A generalized structural equation model tested the mediating role of family resilience in this relationship. Among adolescents aged 12–17 years, 34.22% were reported to have experienced MEDBH condition. Adolescents with one or more ACEs had significantly higher odds of experiencing MEDBH issues (aOR = 2.59, 95% CI: 2.56–2.96), while family resilience was protective (aOR = 0.78, 95% CI: 0.66–0.92), after adjusting for sociodemographic characteristics. Mediation analysis confirmed that family resilience partially mediated the association between ACEs and MEDBH outcomes (β = 0.0100, *p* < 0.001). Specifically, ACEs were negatively associated with family resilience. Our findings emphasize the significant negative association of ACEs on adolescent MEDBH, both directly and indirectly. While family resilience may serve as a protective factor, it is weakened by ACEs. Enhancing family resilience may mitigate the adverse consequences of ACEs.

## Background

Adverse Childhood Experiences (ACEs) are defined as stressful or traumatic events during childhood, including abuse, neglect, and household dysfunction, that have long-term consequences on an individual’s physical and mental health (Felitti et al., 1998; Gilbert et al., 2015; Hughes et al., 2017). Research has consistently linked ACEs to a wide range of adverse health outcomes, including depression, substance use, chronic diseases, and early mortality (Anda, 1999; Bethell et al., 2014; Brown et al., 2009; Kasehagen et al., 2018; Liu et al., 2020). The original ACEs study by the CDC and Kaiser Permanente in 1998 established a strong dose-response relationship between childhood adversity and numerous health complications in adulthood, such as cardiovascular disease, cancer, and psychiatric disorders (Felitti et al., 1998). More recent studies have expanded these findings, highlighting that individuals with multiple ACEs are at higher risk for sedentary behavior, obesity, high-risk behaviors (e.g., smoking, drug use), and poor academic and work performance (Felitti et al., 1998; Ortiz, 2019).

The COVID-19 pandemic exacerbated existing mental health challenges among adolescents, leading to increased rates of depression, anxiety, and suicidal ideation due to social isolation, academic disruptions, and family stress (Anderson et al., 2022; Rosenthal & Thompson, 2020). However, this crisis has continued to worsen post-pandemic, with nearly one in five U.S. adolescents (20.1%) experiencing a major depressive episode in 2022, and 14.4% reporting serious thoughts of suicide (Substance Abuse and Mental Health Services Administration [SAMHSA], 2023). Several factors contribute to this growing crisis, including heightened academic pressures, increased social media exposure, economic instability, and barriers to mental health care (SAMHSA, 2023). Limited access to professional mental health services has further compounded these issues, leaving many adolescents without adequate support (SAMHSA, 2023).

Given the rising burden of mental health disorders among youth, identifying modifiable protective factors to mitigate the long-term effects of ACEs is crucial. Increasing attention has focused on family resilience and its role in helping individuals cope with adversity and protect mental health (Prime et al., 2020; Ungar & Theron, 2020). Defined as the ability of families to adapt to and recover from adversity (Gómez et al., 2024; Ungar, 2016; Walsh, 2016), family resilience has emerged as a key determinant of adolescent well-being. Strong family resilience, characterized by effective communication, conflict resolution, and adaptive coping, has been associated with reduced risks of depression and behavioral disorders, as well as improved physical health outcomes such as lower rates of obesity and hypertension (Barnhart et al., 2022; Gouin et al., 2017; Hall et al., 2021; Liu et al., 2020).

Despite growing recognition of resilience as a protective factor, its role as a mediating mechanism in the relationship between ACEs and mental, emotional, developmental, or behavioral health (MEDBH) outcomes remains poorly understood. Previous studies have examined family resilience as a mediator between ACEs and specific health outcomes, such as insufficient sleep (Baiden et al., 2024) and young children’s flourishing (Thompson et al., 2024), but these investigations often focus on singular health aspects or younger populations. Additionally, while resilience has been studied in the context of ACEs (Lackova Rebicova et al., 2021; Wang et al., 2023), few studies have specifically examined family resilience as a distinct mediating factor in adolescent MEDBH outcomes. In summary, most prior research has either assessed resilience broadly or explored its effects on individual conditions, limiting a full understanding of its role in adolescent MEDBH.

To address these gaps, our study used a nationally representative sample to investigate the mediating role of family resilience in the relationship between ACEs and adolescent MEDBH outcomes among children aged 12 to 17.

## Methods

### Outcome Variable

#### Mental Emotional Developmental or Behavioral Health (MEDBH) 

The MEDBH variable was measured based on parental or caregiver reports indicating whether their child currently experienced at least one of ten listed health conditions. These conditions included Tourette syndrome, anxiety disorders, depression, behavioral or conduct disorders, developmental delays, intellectual disabilities, speech or language disorders, learning disabilities, autism spectrum disorder (ASD), and attention-deficit disorder or attention-deficit/hyperactivity disorder (ADD/ADHD). The variable was coded as a binary categorical measure, where Yes = the presence of one or more conditions, and No = the absence of any listed conditions.

### Independent Variables

#### Family Resilience 

Family resilience was measured using four items assessing how often family members talked together, worked together, drew on strengths, and remained hopeful. The composite variable was dichotomized in the NSCH dataset as *Family Demonstrates Resilience* and *Family Does Not Demonstrate Resilience*.

#### Adverse Childhood Experiences (ACEs) 

The ACEs variable was measured using eleven items that assessed whether the child had experienced parent or guardian divorce, parent or guardian death, parent or guardian incarceration, adults slapping, hitting, kicking, or punching others, witnessing violence, living with someone with a mental illness, living with someone who has an alcohol or drug problem, being treated unfairly due to race, being treated unfairly because of sexual orientation or gender identity, being treated unfairly due to a health condition, and facing hardships in securing food and housing. A composite ACEs score was calculated based on the total number of ACEs reported, and a categorical variable was derived to distinguish between children who had experienced at least one ACEs (*one or more ACE*) and those who had not (*no ACEs*).

#### Covariates

This consisted of variables Age (12-17years); Sex (Male/Female); Race (White, Black or African American, Asian, Others, Two or more); Ethnicity (Not Hispanic/Hispanic); Household Income (0–99% FPL, 100–199% FPL, 200–399% FPL, 400% FPL+); Employment of at least one parent (Not Employed/Employed) and Health Insurance Status (Insured/Uninsured).

### Statistical Analysis

This study utilized weighted cross-sectional data from the 2022 National Survey of Children’s Health (NSCH) to examine associations between ACEs, family resilience, and MEDBH outcomes among adolescents aged 12–17 years. Descriptive statistics were calculated to summarize sample characteristics, followed by chi-square tests to examine group differences by MEDBH status. Multivariable logistic regression models assessed the independent and combined associations of ACEs and family resilience with MEDBH outcomes, adjusting for sociodemographic covariates, including age, sex, race/ethnicity, income, parental employment, and insurance status. A generalized structural equation model (GSEM) was used to estimate both direct and indirect effects of ACEs on MEDBH outcomes through family resilience. All data analyses were conducted using STATA version 17.

## Results

Table 1 shows the sociodemographic characteristics of study respondents in the 15–17 age group. The sex distribution was nearly balanced, with a slight predominance of males (51.91% male vs. 48.09% female). Over three-quarters of the sample were White, and more than 80% were not of Hispanic ethnicity. Most participants had at least one employed parent (81.91%) and came from families with household incomes at or above four times the Federal Poverty Level (42.11%). Nearly 95% of respondents had some form of health insurance.

**Table 1:** Descriptive characteristics of the NSCH study sample children aged 12–17 years (*N* = 19,028)

	Count (*N*)	Percent (%)	Presence of MEDBH *N* (%)	Absence of MEDBH *N* (%)
Age (years)**				
12	2,625	13.80	842 (12.93)	1783 (14.25)
13	2,787	14.65	874 (13.42)	1913 (15.28)
14	3,011	15.82	1,025 (15.74)	1986 (15.87)
15	3,341	17.56	1,148 (17.63)	2193 (17.52)
16	3,518	18.49	1,238 (19.01)	2280 (18.22)
17	3,746	19.69	1,385 (21.27)	2361 (18.86)
Sex**				
Male	9,878	51.91	3,242 (49.79)	6636 (53.02)
Female	9,150	48.09	3,270 (50.21)	5880 (46.98)
Race**				
White	14,489	76.15	5,227 (80.27)	9262 (74.00)
Black or African American	1,495	7.86	433 (6.65)	1062 (8.49)
Asian	1,271	6.68	183 (2.81)	1088 (8.69)
Others	359	1.89	129 (1.98)	230 (1.84)
Two or more Races	1,414	7.43	540 (8.29)	874 (6.98)
Ethnicity (Hispanic)				
Not Hispanic	16,007	84.12	5,569 (85.52)	10,438 (83.40)
Hispanic	3,021	15.88	943 (14.48)	2,078 (16.60)
Household Income**				
0–99% FPL	2,467	12.97	907 (13.93)	1560 (12.46)
100–199% FPL	3,107	16.33	1,117 (17.15)	1990 (15.90)
200–399% FPL	5,442	28.60	1,876 (28.81)	3566 (28.49)
400% FPL+	8,012	42.11	2,612 (40.11)	5400 (43.14)
Employment**				
Not Employed	3,322	18.09	1,366 (21.62)	1,956 (16.25)
Employed	15,037	81.91	4,953 (78.38)	10,084 (83.75)
Health Insurance Status **				
Insured	18,066	94.94	6,262 (96.16)	11,804 (94.31)
Uninsured	833	4.38	223 (3.42)	610 (4.87)
MEDBH				
No MEDBH issue	12,516	65.78		
MEDBH issue	6,512	34.22		

** *p*-value <.001

The overall prevalence of MEDBH issues among the sample was 34.22%. Table 2 presents three logistic regression models predicting the presence of MEDBH issues, utilizing ACEs (Model 1), family resilience (Model 2), and both (Model 3) as predictors while adjusting for sociodemographic characteristics. In Model 1, adolescents with one or more ACEs had significantly higher odds of experiencing MEDBH issues (aOR = 2.63, 95% CI: 2.31–3.00) compared to those with no ACEs exposure. In Model 2, adolescents from families demonstrating resilience had 30% lower odds of experiencing MEDBH issues compared to those from families not demonstrating resilience (aOR = 0.70, 95% CI: 0.60–0.83). In Model 3, which included both ACEs and family resilience, the association between ACEs and MEDBH issues remained strong (aOR = 2.59, 95% CI: 2.56–2.96), and the protective effect of family resilience continued to be significant (aOR = 0.78, 95% CI: 0.66–0.92).

**Table 2:** Logistic regression models predicting MEDBH with adverse childhood experiences (ACEs), family resilience, and both ACES and family resilience as predictors after controlling for demographic variables using National children’s health survey (NSCH) data

	Model 1^#^	Model 2^#^	Model 3^#^
Variables	aOR (95% CI)	aOR (95% CI)	aOR (95% CI)
ACE			
No ACE	Ref	-	Ref
One or more ACE	2.63**(2.31–3.00.31.00)		2.59**(2.56–2.96)
Family Resilience			
*Does Not Demonstrate Resilience*		Ref	Ref
*Demonstrates Resilience*		0.70**(0.60–0.83)	0.78**(0.66–0.92)

*p < 0.05, **p < 0.01

^#^Model adjusted for age, sex, race, Ethnicity, income level, parent’s employment status, and health insurance coverage

**Fig. 1:**
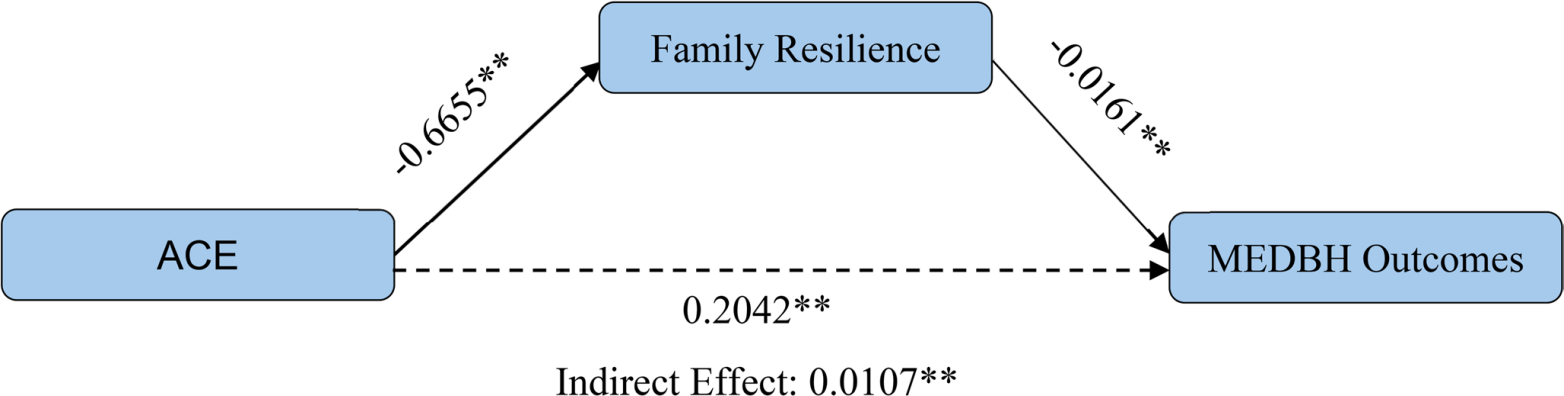
Mediation Model

Using a generalized structural equation model, we found that ACEs had both direct and indirect effects on MEDBH) outcomes through family resilience (Fig. 1). Adolescents with one or more ACEs were more likely to experience MEDBH issues (β = 0.2042, *p* < 0.001). ACEs were negatively associated with family resilience (β = −0.6655, *p* < 0.001). Additionally, family resilience was negatively associated with MEDBH (β = −0.0161, *p* < 0.001), meaning that higher family resilience is associated with lower MEDBH outcomes.

The significant indirect effect or mediation effect, calculated as (β = [(−0.6655) X (−0.0161)] = 0.0107, *p* < 0.001), confirms that family resilience partially mediates the relationship between ACEs and MEDBH outcomes.

In summary, higher levels of ACEs directly increase the likelihood of MEDBH outcomes, while ACEs also indirectly worsen MEDBH outcomes by diminishing the protective effect of family resilience.

## Discussion

The primary aim of our study was to explore the complex interplay of relations between ACEs, family resilience, and MEDBH outcomes among adolescents aged 12–17. Our study results showed that adolescents who had experienced ACEs had worse MEDBH outcomes. However, family resilience mediated the impact of ACEs on MEDBH, meaning that adolescents with higher ACEs tend to have lower family resilience, which in turn worsened MEDBH outcomes.

### ACEs and MEDBH

Findings from this study reinforce the well-documented association between ACEs and poor mental health outcomes (Gilbert et al., 2015; Hall et al., 2021; Hughes et al., 2017). Furthermore, this link has been shown to endure across the lifespan (Bethell et al., 2014; Kasehagen et al., 2018; Liu et al., 2020; Ortiz, 2019). Our results revealed that adolescents who experienced ACEs were more than 2.5 times as likely to report negative MEDBH outcomes compared to those without such experiences. Unlike prior studies that primarily focused on specific mental health disorders, the present study employed a more comprehensive assessment of mental health outcomes, encompassing developmental, behavioral, and emotional dimensions. This broader approach allowed for a more nuanced understanding of how ACEs influence mental health across multiple domains.

The mechanisms underlying this association of ACEs and MEDBH are multifaceted. ACEs can trigger chronic stress responses, leading to dysregulation of emotional and behavioral coping systems. Adolescents can adopt maladaptive coping behaviors such as substance use, overeating, or risky sexual activity that increase the likelihood of long-term health consequences (Felitti et al., 2019). Additionally, youth with ACEs exposure have been shown to be significantly more likely to experience poverty, felony charges, early parenthood, and reduced educational attainment that might lead to adverse mental health outcomes (Giovanelli et al., 2016; Ratcliff et al., 2025). The effects of ACEs have also been reported to extend intergenerationally; children residing in households with adults who have high ACEs exposure are nearly three times more likely to exhibit significant mental health impairments compared to children in low-ACEs households (Decker et al., 2024). Children exposed to multiple ACEs are also at significantly higher risk of developmental delays, with studies showing a dose-response relationship (Nivens et al., 2024). Collectively, these findings highlight the necessity for early intervention and the implementation of trauma-informed care strategies in both clinical and community contexts to reduce the comprehensive mental health impacts of ACEs.

### Family Resilience and MEDBH

Our study offers a distinctive perspective by evaluating resilience not solely as an individual trait but as a collective family dynamic, emphasizing communication, collaboration, and optimism among family members. Effective communication within the family unit fosters a secure and supportive environment, enabling children to express their emotions and navigate challenging situations more effectively, thereby promoting optimal development and mental health (Bethell et al., 2019). Additionally, family engagement in shared activities, such as communal meals and recreational play, has been linked to better-adjusted children, highlighting the significance of collaborative family practices in nurturing child well-being (Bilodeau et al., 2023). Optimism within the family plays a critical role in shaping children’s optimism and psychological well-being. Empirical evidence suggests that parental optimism, particularly when demonstrated during challenging circumstances, serves as a powerful social model from which children learn adaptive coping strategies (Qi et al., 2022). Adolescents with higher levels of optimism are more likely to engage in health-promoting behaviors (Malinowska-Cieślik et al., 2019) and display greater psychological well-being (Krok & Telka, 2019). These findings emphasize the importance of fostering positive family interactions and attitudes to support children’s mental health and overall development.

### Indirect Effect of Family Resilience on the Relationship between ACEs and MEDBH

The findings of the present study show the critical mediating role of family resilience in the association between ACEs and MEDBH outcomes among adolescents. Consistent with prior research, we observed that greater exposure to ACEs is significantly associated with reduced levels of family resilience, which in turn amplifies the risk of poor mental health outcomes (Elmore et al., 2020; Heard-Garris et al., 2018; Schneider et al., 2019). Similarly, prior work has demonstrated that positive parental behaviors, such as affectionate parent-child interactions, could partially mediate the effects of ACEs on developmental delays (Bellis et al., 2017; Webster, 2022).

Family resilience, conceptualized as a dynamic process encompassing positive adaptation, open communication, and emotional cohesion within the family unit, serves as a protective factor against the deleterious psychological effects of early adversity (Uddin et al., 2020). Adolescents embedded in resilient family systems are more likely to benefit from emotional security, psychological support, and coping strategies that facilitate the processing of stress and adversity. These youth report fewer symptoms of depression and anxiety and exhibit more adaptive functioning compared to peers lacking such support (Bethell et al., 2019; Thompson et al., 2024; Ungar, 2015). Family-based strengths such as shared optimism and unity in the face of challenges foster a supportive environment where children learn to manage emotions, express themselves, and internalize hope. In contrast, the absence of such support and the presence of ongoing household dysfunction have been linked to heightened vulnerability to psychiatric conditions such as depression, anxiety, and conduct disorders (Uddin et al., 2020).

Importantly, these protective processes can also help disrupt the intergenerational transmission of trauma, a phenomenon in which the effects of unaddressed adversity and stress in one generation are passed on to the next through parenting behaviors, environmental stressors, and epigenetic mechanisms (Yehuda & Lehrner, 2018). Families that exhibit resilience are better equipped to protect children from the psychological and behavioral consequences of inherited trauma. This occurs through intentional emotional responsiveness, development of secure attachment relationships, and fostering of open dialogue around past hardships (Heard-Garris et al., 2018). By facilitating emotional regulation, reinforcing positive identity development, and promoting consistent caregiving, family resilience serves as a transformative force capable of breaking entrenched cycles of mental health vulnerability (Gartland et al., 2019; Walsh, 2016). Taken together, these findings affirm the importance of incorporating family-centered approaches in interventions aimed at mitigating the long-term impact of ACEs on overall adolescent mental and developmental health, with family resilience serving as a key target for prevention and healing.

### Limitations and Strengths

This study uses a cross-sectional design that limits us from establishing causality. While we can identify a strong association between family resilience and improved MEDBH, we cannot conclude that family resilience directly causes these improvements. However, the study uses nationally representative samples, allowing the findings drawn from the study to be generalized to diverse adolescent populations in the US. The large sample size also makes the results of the study more reliable. Furthermore, the study uses self-reported survey data, which may lead to recall bias due to people having difficulty recalling information and misinterpreting the questions. Since the study utilizes secondary data, the questions in the dataset are limited, and new variables such as individual-level resilience cannot be measured, which could provide deeper insights into the research. Nevertheless, unlike other studies that only focus on a single mental health outcome, this study looks at various mental, emotional, developmental, and behavioral indicators in adolescents between the ages of 12 and 17 years, where MEDBH is commonly seen. Finally, our study uses SEM to understand the pathways linking the risk factors for MEDBH, which allows for an in-depth examination, strengthening the reliability of the findings.

## Conclusion

In conclusion, the study offers important insights into how family resilience can shape the MEDBH of adolescents who have faced ACEs. Recognizing the role of family resilience in potential interventions extending beyond individual-level support, and focusing on the strength and adaptability of the family unit. Supporting families in strengthening communication, collaboration, shared problem-solving, and a sense of hope may be a critical step toward promoting long-term well-being for adolescents.

## Data Availability

Data used in the study is publicly available for download at https://www.childhealthdata.org/learn-about-the-nsch/NSCH
